# Dual Role of Hepatic Macrophages in the Establishment of the *Echinococcus multilocularis* Metacestode in Mice

**DOI:** 10.3389/fimmu.2020.600635

**Published:** 2021-01-08

**Authors:** Hui Wang, Chuan-Shan Zhang, Bin-Bin Fang, Jiao Hou, Wen-Ding Li, Zhi-De Li, Liang Li, Xiao-Juan Bi, Liang Li, Abuduaini Abulizi, Ying-Mei Shao, Ren-Yong Lin, Hao Wen

**Affiliations:** ^1^ State Key Laboratory of Pathogenesis, Prevention and Treatment of High Incidence Diseases in Central Asia, Clinical Medicine Institute, The First Affiliated Hospital of Xinjiang Medical University, Urumqi, China; ^2^ Basic Medical College, Xinjiang Medical University, Urumqi, China; ^3^ Branch of The First Affiliated Hospital of Xinjiang Medical University, Changji, China; ^4^ Xinjiang Key Laboratory of Echinococcosis, Clinical Medicine Institute, WHO Collaborating Centre on Prevention and Case Management of Echinococcosis, The First Affiliated Hospital of Xinjiang Medical University, Urumqi, China; ^5^ Chronic Disease Laboratory, Institutes for Life Sciences and School of Medicine, South China University of Technology, Guangzhou, China; ^6^ Department of Hepatic Hydatid and Hepatobiliary Surgery, Digestive and Vascular Surgery Centre, The First Affiliated Hospital of Xinjiang Medical University, Urumqi, China

**Keywords:** Alveolar echinococcosis, *Echinococcus multilocularis*, inflammation response, hepatic macrophages, M1/M2 macrophages

## Abstract

*Echinococcus multilocularis* larvae, predominantly located in the liver, cause a tumor-like parasitic disease, alveolar echinococcosis (AE), that is characterized by increased infiltration of various immune cells, including macrophages, around the lesion that produces an “immunosuppressive” microenvironment, favoring its persistent infection. However, the role of hepatic macrophages in the host defense against *E. multilocularis* infection remains poorly defined. Using human liver tissues from patients with AE and a hepatic experimental mouse model of *E. multilocularis*, we investigated the phenotype and function of hepatic macrophages during the parasite infection. In the present study, we found that a large number of CD68^+^ macrophages accumulated around the metacestode lesion in the liver of human AE samples and that both S100A9^+^ proinflammatory (M1 phenotype) and CD163^+^ anti-inflammatory (M2 phenotype) macrophages were significantly higher in close liver tissue (CLT) than in distant liver tissue (DLT), whereas M2 macrophages represent the dominant macrophage population. Furthermore, *E. multilocularis*-infected mice exhibited a massive increase in macrophage (F4/80^+^) infiltration in the liver as early as day 5, and the infiltrated macrophages were mainly monocyte-derived macrophages (CD11b^hi^ F4/80^int^ MoMFs) that preferentially differentiated into the M1 phenotype (iNOS^+^) at the early stage of *E. multilocularis* infection and then polarized to anti-inflammatory macrophages of the M2 phenotype (CD206^+^) at the chronic stage of infection. We further showed that elimination of macrophages by treatment of mice with clodronate-liposomes before *E. multilocularis* infection impaired worm expulsion and was accompanied by a reduction in liver fibrosis, yielding a high parasite burden. These results suggest that hepatic macrophages may play a dual role in the establishment and development of *E. multilocularis* metacestodes in which early larvae clearance is promoted by M1 macrophages while persistent metacestode infection is favored by M2 macrophages.

## Introduction

Alveolar echinococcosis (AE) is one of the most dangerous parasitic diseases distributed in the Northern Hemisphere and is caused by the larval stage of the tapeworm *Echinococcus multilocularis* (*E. multilocularis*) (1–4). *E. multilocularis* larvae are transported *via* the portal vein blood flow to the liver and predominantly dwell in the liver parenchyma, where they develop into a metacestode with subsequent infiltrative growth similar to a malignant tumor, gradually destroy the surrounding host tissues (5) and are able to spread to any organ through local invasion and metastases (6–8). Parasitic invasion is generally asymptomatic for a long period, as diagnosed patients usually have reached an advanced stage and have a mortality rate of 75% to 90% if untreated within 10~15 years (9, 10). Although great progress has been made in the field of hepatic AE surgery, including radical resection, liver transplantation or *ex vivo* liver resection and autotransplantation, with promising clinical outcomes (11), there are still some patients with AE who are not eligible for surgery treatment and lose the chance to save their life. Thus, there is an urgent need to clarify the pathogenic mechanisms of this lethal disease and to develop novel therapeutic approaches for AE.

The intrahepatic localization and persistent proliferation of *E. multilocularis* usually elicit a severe hepatic granulomatous inflammation response characterized by attraction of various host immune cells around the metacestode lesion (12), which may produce an “immunosuppressive” microenvironment and might be of crucial importance in favoring persistent *E. multilocularis* infection. Recent histological analysis of the liver sections of patients with AE has shown that the infiltrated immune cells were composed of large clusters of T lymphocytes (predominantly CD8^+^ T cells), as well as macrophages with an epithelium-like arrangement, mainly located in close contact with the laminated layer of the parasitic vesicle (12, 13). Studies in a mouse experimental model of intrahepatic *E. multilocularis* infection also showed that increased macrophages were scattered in the granuloma as early as day 21 and progressively exceeded the infiltrated T lymphocytes. At the late stage, similar to observations in human AE, macrophages were mainly located at the periphery of the lesion (14). These observations in humans and experimental studies in mice indicated that macrophages are among the main infiltrated cell population and may act as key immune regulators of the granulomatous inflammation response to maintain immune homoeostasis during persistent infection (15, 16). However, the role of macrophages in the development of *E. multilocularis* larvae has not yet been fully elucidated.

Multiple lines of evidence point to macrophages being markedly heterogeneous immune cells because they are ontologically and functionally diverse. In the liver, macrophages consisting of resident Kupffer cells (KCs), which originate from fetal yolk sacks, and recruited monocyte-derived macrophages (MoMFs), which originate from bone marrow-derived monocytes. KCs play a major role in maintaining immunological tolerance and provide an anti-inflammatory micromilieu during homeostasis in the liver. MoMFs are massively attracted to inflammatory sites and represent the dominant macrophage population during acute or chronic injury to the liver, which regulates both the initial inflammatory response and the late healing response (17). It has been shown that macrophages accumulate at the lesion site during *E. multilocularis* infection, but the origin of these accumulated macrophages has not been definitively demonstrated. In addition, the functional diversity of macrophages relates to their ability to polarize into different functional phenotypes. On the basis of Th1/Th2 polarization concepts, macrophages can be polarized into proinﬂammatory M1 macrophages and anti-inﬂammatory M2 macrophages (18). M1 macrophages are induced by Th1-type cytokines (such as IFN-γ) and are characterized by upregulation of the expression of nitric oxide synthase (iNOS) and exhibit strong microbicidal and tumoricidal properties. In contrast, M2 macrophages are induced by Th2-type cytokines (such as IL-4 or IL-13) (19, 20) and are characterized by upregulation of the expression of mannose receptor (also known as CD206), Ym1, and Fizz1 and secretion of immune-modulatory mediators (such as IL-10 and TGF-β) (21), which are supposed to contribute to parasite infestation, tissue remodeling, and tumor progression (22). Coincidentally, previous studies have shown that the host initially responds to *E. multilocularis* infection with an acute inflammatory Th1-biased immune response, but this response gradually shifts to Th2-oriented immune suppression responses at the progressive phase of AE (23–27). Thus, we speculate that M1/M2 macrophage polarization may occur accompanied by the Th1/Th2 response during *E. multilocularis* infection. Previous studies in some other parasites, such as *Trypanosoma cruzi* (28), *Fasciola hepatica* (29), and *Schistosoma japonicum* (30), have shown that M2 macrophages are induced to escape being killed by M1 macrophages and prolong infection. Therefore, whether *E. multilocularis* has the ability to switch from proinflammatory M1 at the early stages of infection to an anti-inflammatory M2 macrophage during the advanced stages of infection to maintain its persistent infection needs to be clarified.

In the present study, we investigated the heterogeneity of hepatic macrophages in both human liver tissues from patients with AE and a quantitative hepatic experimental mouse model of *E. multilocularis*. We further evaluated the role of hepatic macrophages in the establishment of *E. multilocularis* metacestode using a novel clodronate-liposome (CL) injection regime, which can effectively eliminate macrophages. Our results demonstrated that macrophages are readily recruited to the parasite lesion site and predominantly exhibit an M2 phenotype in both human AE and mouse models. In addition, we found that the early expulsion ability of *E. multilocularis* larvae was impaired after depletion of hepatic macrophages, which was accompanied by inhibition of liver fibrosis formation and facilitated *E. multilocularis* metacestode growth. This led to a high parasite burden. Our results suggest that hepatic macrophages play an important role in the *E. multilocularis* metacestode establishment and development.

## Materials and Methods

### Human Subjects and Liver Immunohistochemistry

Patients with AE included in this study were diagnosed with ultrasound (US), computed tomography (CT) and biopsy-proven AE in the First Affiliated Hospital of Xinjiang Medical University, Urumqi, China. Liver tissue samples were obtained from patients who underwent surgical resection, and the liver tissue standards were taken according to a previous study: two liver tissue samples were prepared from each patient with AE, which were close liver tissue (CLT) and distant liver tissue (DLT) from the lesion (13). This study was conducted under the ethics approval of the First Affiliated Hospital of Xinjiang Medical University (S20130418-3), with all patients having given informed consent.

Paraformaldehyde-fixed and paraffin-embedded liver tissue slices, 4 μm thick, were deparaffinized and rehydrated and then processed for hematoxylin and eosin (H&E) and immunohistochemical (IHC) staining. For IHC analysis, slices were subjected to heat-induced antigen retrieval using citric acid buffer (ZSGB-BIO, Beijing). After the slices cooled to room temperature (RT), endogenous peroxidase activity was blocked with 3% H_2_O_2_, and nonspecific binding was blocked with 10% normal serum with 1% BSA in tris-buffered saline (TBS) for 1 h at RT. Slices were then incubated with primary antibodies (CD68, 1:100, ab955; CD163, 1:500, ab182422; S100A9, 1:1000, ab63818, Abcam, Cambridge, UK) at 4°C overnight. The next day, slices were rinsed three times with tris-buffered saline and 0.1% Tween (TBST) for 15 min and then incubated with the secondary antibody for 2 h at RT. Visualization was induced with a diaminobenzidine (DAB) substrate kit (ab64238, Abcam, Cambridge, UK) according to the manufacturer’s instructions. For all immunoreactions, negative controls (the primary antibody was replaced with preimmune serum or TBS) were also included. Representative images from H&E and IHC-stained sections were captured using a digital image-capture system (Olympus, Tokyo) at a magnification between 100× and 400×. For quantitation, staining was then assessed at 400× magnification in a total of 3–5 fields/slice/liver sample using the cellSens Dimension software (Olympus), and the results were expressed as the percentage of positive area to the total measured area.

### Mice

Pathogen-free female C57BL/6 wild-type mice (6 weeks of age) were purchased from Beijing Vital River Experimental Animal Technology Co. Ltd. The animals were housed in a specific pathogen–free environment with 12-h light/dark cycles and provided rodent chow diet and water *ad libitum* at the Animal Facility of Xinjiang Medical University. All mice received humane care in compliance with the Medical Research Center’s guidelines, and all experimental protocols involving mice were approved by the Ethics Committee of the First Affiliated Hospital of Xinjiang Medical University (Approval No. 20170809-01). All surgery was performed under chloral hydrate anesthesia, and all efforts were made to minimize suffering.

### Parasites and Mouse Model of *Echinococcus multilocularis* Infection

*E. multilocularis* protoscoleces (PSCs) were obtained from intraperitoneal lesions maintained in BALB/c mice under aseptic conditions (31). Only PSCs exhibiting over 95% vitality were counted and subsequently used to infect experimental mice. To establish the hepatic experimental *E. multilocularis* infection mouse model, each mouse was inoculated through the hepatic portal vein with different doses of PSCs in RPMI 1640 medium (Gibco, Auckland, New Zealand) to induce a quantitative hepatic infection model. According to the inoculation numbers of PSCs, the experimental mice were divided into low dose group (LD, 50 PSCs), medium dose group (MD, 500 PSCs), and high dose group (HD, 2000 PSCs), whereas control group mice were injected with the same volume of RPMI 1640, as described previously (32). For flow cytometry analysis, five to six mice in LD, MD, HD, and control group were sacrificed at 2, 12, and 24 weeks to characterize the composition and phenotype of the hepatic macrophages. For histopathological analysis, five to six mice in HD group (2000 PSCs, a clear infectious dose) (32) were sacrificed at 2, 5, 8, 11 days, 2, 12, and 24 weeks, respectively. The liver surface was carefully screened for hepatic lesions for a preliminary assessment of the intensity of *E. multilocularis* infection and then the whole liver tissue samples were collected from the mice and taken for further analysis at each time point.

### *In Vivo* Depletion of Hepatic Macrophages

Administration of clodronate liposomes (CL) to mice were currently the most effective and widely used method to deplete macrophages *in vivo* (33). To determine an appropriate administration protocols of CL, mice were injected intravenously (i.v.) and intraperitoneally (i.p.) with CL (Liposoma BV, AMSTERDAM, Netherlands; 100 µl of suspension/10 grams of animal weight) respectively to evaluate the deplete efficiency of hepatic macrophage at 1, 3, and 7 days by flow cytometry analysis.

### Histopathological and Immunohistochemical Analyses of Mouse Liver Tissues

For histopathological analysis, all liver lobes were divided into three sections and fixed in 4% (v/v) paraformaldehyde in PBS, embedded in paraffin, cut into 4-μm-thick slices and subjected to H&E staining to examine inflammatory cell infiltration and changes in general histology. The number of “infectious foci” with PSCs or parasitic vesicles was counted from all liver lobes under a microscope (Olympus, Tokyo) after H&E staining. In addition, the lesion areas (μm^2^) in 10–15 fields/slice/mouse (40×) and the thickness (μm) of the infiltration zone of inflammatory cells surrounding the parasitic lesion with identical germinal layers were measured by computer-assisted morphometric analysis using cellSens Dimension software (Olympus, Tokyo, Japan) for macrophage depletion experiments.

For IHC analysis, slices were examined to determine the expression and distribution of F4/80 (1:100, ab6640; Abcam, Cambridge, UK), CD4 (1:100, 14-9766-82; eBioscience), and α-SMA (1:500, ab 124964; Abcam) at each time point. Sirius red (SR) staining was also performed for fibrosis analysis. Slices were then examined and photographed at 100×, 200×, and 400× magnification using a digital image-capture system (Olympus, Tokyo, Japan). The area with intensely positive staining was measured at 200× magnification in a total of 3–5 fields/section/sample using computer-assisted morphometric analysis in cellSens Dimension software (Olympus, Tokyo, Japan). Data were presented as the percentage of positive area to the total measured area.

For immunofluorescence analysis, liver tissues were fixed in 4% paraformaldehyde, cryoprotected with 30% sucrose solution, then embedded in OCT, and sectioned at a thickness of 6 μm. Dual immunofluorescence for F4/80 (1:100, ab6640; Abcam) and CCR2 (1:100, ab203128; Abcam) was performed on frozen slices at 4°C overnight followed by incubation with Alexa Fluor^®^ 488-labeled anti-rabbit IgG (Cell Signaling Technology, Boston, MA; cat. no. 4412S) and DyLight^®^ 594-labeled anti-rat IgG (ab102224; Abcam) at RT for 2 h; then, slices were mounted in DAPI mounting medium (ab104139; Abcam) for nuclear staining. Images were acquired by a confocal laser scanning fluorescence microscope (Leica, Oskar-Barnack-Straße).

### Hepatic Nonparenchymal Cell Isolation of Mouse Liver Tissues

Hepatic nonparenchymal cells (HNPCs) were isolated with reference to previous studies (34, 35). Briefly, mouse livers *in situ* were sequentially perfused with EGTA (Sigma-Aldrich, cat. no. E4378), pronase E (5 mg/ml; Sigma-Aldrich, cat. no. P5147), and collagenase type I (0.5 mg/ml; Worthington Biochemical Corporation, Lakewood, USA, cat. no. LS004197) solution through the portal vein followed by cutting of the inferior vena cava to remove circulating cells. Then, the digested livers were harvested and homogenized thoroughly and further digested with prewarmed pronase E/collagenase I solution containing 1% (vol/vol) DNase I (Roche, Indianapolis, USA; cat. no. 10-104-159-001) for 20 min at 37°C in a shaker (120 rpm). The resulting cell suspension was filtered through a 70-μm cell strainer and centrifuged two times (50 × g, 5 min, 4°C) to remove hepatic parenchymal cells. The remaining HNPCs containing hepatic macrophages were harvested by centrifugation at 500 × g for 10 min and enriched by density gradient–mediated separation (800 × g, 20 min, 20°C) over a 25/70% Percoll gradient (GE Healthcare). Then, the HNPCs were collected by removing the cell layer from the gradient interface, washed with DMEM containing 10% FBS (Gibco, Auckland, New Zealand), harvested by centrifugation at 500 × g for 10 min at 4°C, resuspended, counted, and used for flow cytometry analysis.

### Flow Cytometry

Nonspecific antibody binding was blocked by incubating the isolated HNPCs with purified anti-CD16/CD32 antibody (Fc Block; BioLegend, San Diego, CA) for 20 min at 4°C followed by incubation with combinations of surface antibodies for 30 min at 4°C in the dark. For intracellular flow cytometry, cells were then fixed and resuspended in permeabilization buffer containing intracellular antibodies according to the manufacturer’s instructions (BD Biosciences; cat. no. 554714). Cell preparations were analyzed immediately on an LSRFortessa flow cytometer (BD Immunocytometry Systems, San Jose, CA, USA). Data were analyzed using FlowJo software (version V10; Tree star, Inc., Ashland, OR, USA). Information on the antibodies utilized in this assay is listed in **Supporting Table S1**.

### Quantitative Real-Time PCR

For qRT-PCR analysis, liver tissues were taken from parasitic lesions in *E. multilocularis*-infected mice and control mice as previously described (32). Total RNA was extracted from mouse liver tissue using TRIzol Reagent (Invitrogen, Carlsbad, CA) and reverse transcribed into cDNA according to the manufacturer’s instructions (Thermo Fisher Scientific, Waltham, MA; cat. no. K1622). Then, the cDNA was subjected to qRT-PCR by the SYBR Green PCR premix (TaKaRa, Dalian, China) in a thermocycler (iQ5 Bio-Rad, Hercules, CA) as previously described (34). Primer sequences for the genes analyzed are listed in **Supporting Table S2**. Gene expression was normalized to the housekeeping gene GAPDH. Relative mRNA expression was calculated using the 2^-ΔΔCt^ method.

### Statistical Analysis

Statistical analyses were conducted using GraphPad Prism 6.0 (GraphPad Software, San Diego, CA). The results are presented as the means ± SEM. Student’s t-test (parametric) or the Mann-Whitney U test (nonparametric) was used when only comparing two groups. One-way ANOVA with a Tukey’s multiple comparison was used when there were more than two groups were compared. *P <*0.05 was considered significant in all experiments. (*P*-values were expressed as follows: *P < 0.05; **P< 0.01; ***P < 0.001; ****P< 0.0001).

## Results

### Macrophages Accumulated in the Periphery Areas of the Liver Lesions of Patients With Alveolar Echinococcosis

**Figure 1:**
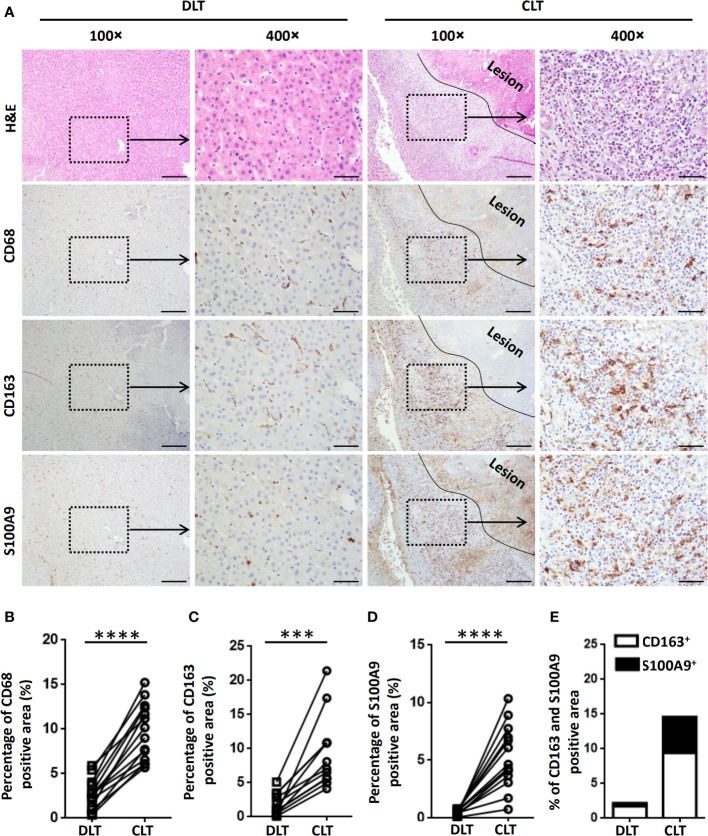
Macrophages accumulate in the peripheral areas of the liver lesions of patients with AE. **(A)** Representative H&E and immunohistochemistry staining for the general macrophage marker CD68, the anti-inflammatory macrophage marker CD163 and the proinflammatory macrophage marker S100A9 in paired liver tissue samples (DLT versus CLT) from patients with AE (scale bar, 200μm for 100× magnification; 50 μm for 400× magnification). The lesion is delimited with a black line. **(B–E)** The percentage of positively stained areas was calculated to assess the expression of CD68, CD163, and S100A9. Close (CLT): “close” liver tissue was approximately 0.5 cm from the lesion (i.e., metacestode); distant (DLT): “distant” liver tissue was at least 2 cm from the lesion. The results are presented as the mean ± SEM (n = 15). ***p < 0.001, ****p < 0.0001.

To investigate the characteristics of hepatic macrophages in patients with AE, we initially examined the expression of CD68 (a marker indicating total macrophages), CD163 (a marker indicating anti-inflammatory M2 macrophages) and S100A9 (a marker indicating proinflammatory M1 macrophages) (15, 36) in AE patient liver specimens by IHC staining. Increased infiltration of CD68^+^ macrophages was readily identified in the inflammatory infiltrate of the periparasitic area of CLT specimens compared with DLT specimens (**Figures 1A, B**). Notably, both proinflammatory S100A9^+^ and anti-inflammatory CD163^+^ macrophages were greatly increased in the periparasitic areas of CLT compared to those in the DLT specimens. However, the increase in the number of CD163^+^ macrophages was larger than that of S100A9^+^ macrophages in the CLT specimens (9.4% of CD163^+^ vs. 5.2% of S100A9^+^), suggesting a predominantly anti-inflammatory response (**Figures 1A, C–E**). Taken together, these findings suggest a role for infiltrating macrophages in human AE and provide a rationale for using animal models to further examine this phenomenon.

### Macrophages Accumulated in the Periparasitic Areas of the Livers of the Mouse Model of *Echinococcus multilocularis*

**Figure 2:**
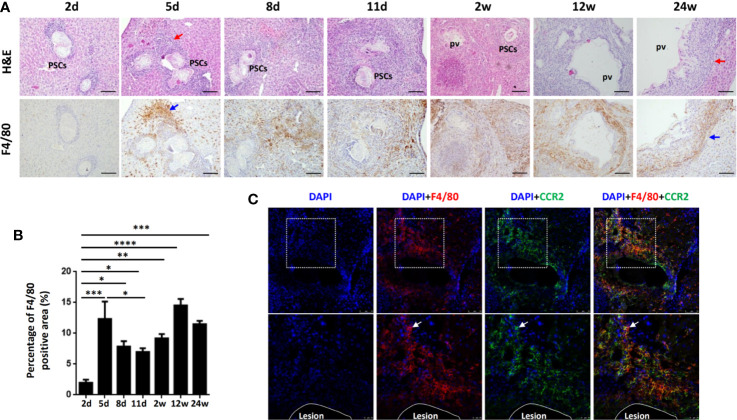
Macrophages accumulate in the inflammatory cell zone around the liver lesions in *E multilocularis*-infected mice during the course of infection. **(A)** Representative H&E staining and F4/80 immunohistochemical staining of liver sections from *E multilocularis*-infected mice at days 2, 5, 8, and 11 and weeks 2, 12, or 24 (200× magnification; scale bar, 100μm). PSCs: protoscoleces. pv: parasitic vesicle. The red arrow indicates the inflammatory cell zone. The blue arrow indicates macrophages. **(B)** Quantification of F4/80^+^ cells. **(C)** Representative images from immunofluorescence staining of DAPI (blue), F4/80 (red), and CCR2 (green) and merged images of liver sections from *E multilocularis*-infected mice at day 5. Higher magnifications of the dotted areas are shown in the lower row. White arrows indicate F4/80 and CCR2 coimmunofluorescence (recruited macrophages). The lesion is delimited with a white line. Data are shown as the mean ± standard error of the mean (SEM, five to six mice per group), *p < 0.05, **p < 0.01, ***p < 0.001, and ****p < 0.0001.

To verify whether macrophage accumulation observed in human AE was evident in the mouse model of *E. multilocularis*, we established a hepatic experimental mouse model of *E. multilocularis* by injecting 2000 PSCs (a clear infectious dose) *via* the portal vein (32). We analyzed F4/80 expression in mouse liver specimens from the early stage (2, 5, 8, 11 days and 2 weeks) to the middle and late stages (12 and 24 weeks) by IHC staining. We found that accompanying the gradual infiltration of lymphocytes around the infectious foci (H&E staining), F4/80^+^ macrophages were significantly increased in the peripheral areas of the infectious foci of *E. multilocularis*-infected mice, markedly increased from day 5 and peaked at week 12 (**Figures 2A, B**). Notably, immunofluorescence costaining for CCR2 (expressed on monocyte-derived cells) and F4/80 at day 5 showed that the accumulated F4/80^+^ macrophages expressed CCR2, indicating that the main source of the accumulating macrophages was largely derived from recruited monocyte-derived macrophages (**Figure 2C**). These results were further confirmed by flow cytometry analysis.

### Dynamic Changes in Hepatic Macrophage Composition During *Echinococcus multilocularis* Infection

**Figure 3:**
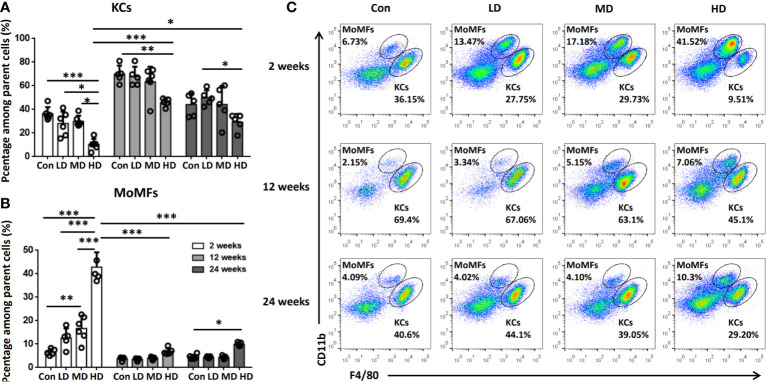
Changes in hepatic macrophage composition in mice infected with different *E multilocularis* PSC inocula during the course of infection. **(A, B)** The percentages of CD11b^int^F4/80^hi^ KCs and CD11b^hi^F4/80^int^ MoMFs in mouse livers infected with different *E multilocularis* PSC inocula during the course of infection. **(C)** Representative flow cytometry plots of hepatic macrophage subsets showing CD11b^hi^F4/80^int^ MoMFs and CD11b^int^F4/80^hi^ KCs; Con; LD: 50 PSCs; MD: 500 PSCs; HD: 2000 PSCs. Data are shown as the mean ± standard error of the mean (SEM, five to six mice per group), *p < 0.05, **p < 0.01 and ***p < 0.001.

To further explore the origin of macrophage accumulation in the livers of mice during *E. multilocularis* infection, we isolated HNPCs from fresh mouse liver tissues infected with different *E. multilocularis* PSC inocula and analyzed them by flow cytometry analysis at 2, 12, and 24 weeks. Total hepatic macrophages were defined as CD45^+^Ly-6G^-^NK1.1^-^CD19^-^CD3^-^CD11b^+^F4/80^+^ cells from the HNPC fraction of the digested liver tissues. Resident KCs were defined as the CD45^+^Ly-6G^-^NK1.1^-^CD19^-^CD3^-^CD11b^int^F4/80^hi^ population, and the recruited MoMFs were defined as the CD45^+^Ly-6G^-^NK1.1^-^CD19^-^CD3^-^CD11b^hi^ F4/80^int^ population (**Supplemental Figure S1**) (34). At 2 weeks after infection, the percentage of KCs was significantly decreased in the livers of the HD group mice compared to MD, LD, or control group mice, whereas there was no significant difference in the absolute numbers of KCs. In contrast, the percentage and absolute numbers of MoMFs were significantly increased in the HD group compared to the MD, LD, and control groups, and even higher than those of KCs in the HD group (**Figures 3A–C**, **Supplemental Figure S2**). At 12 weeks after infection, the percentage of KCs was still decreased in the HD group compared to the control group mice, but the absolute numbers of KCs was increased and higher than that of MoMFs. In contrast, the percentage and absolute numbers of MoMFs in the HD group were decreased and significantly lower than that in the HD group at 2 weeks. At 24 weeks after infection, the percentage of KCs was lower in the HD group than that in the LD and control groups, whereas the percentage of MoMFs was higher in the HD group than that in control group (**Figures 3A–C**, **Supplemental Figure S2**). Taken together, these observations indicate that most hepatic macrophages in infected mice at the early stage arose from monocyte-derived macrophages.

### Dynamic Changes in Hepatic Macrophage Phenotypes During *Echinococcus multilocularis* Infection

**Figure 4:**
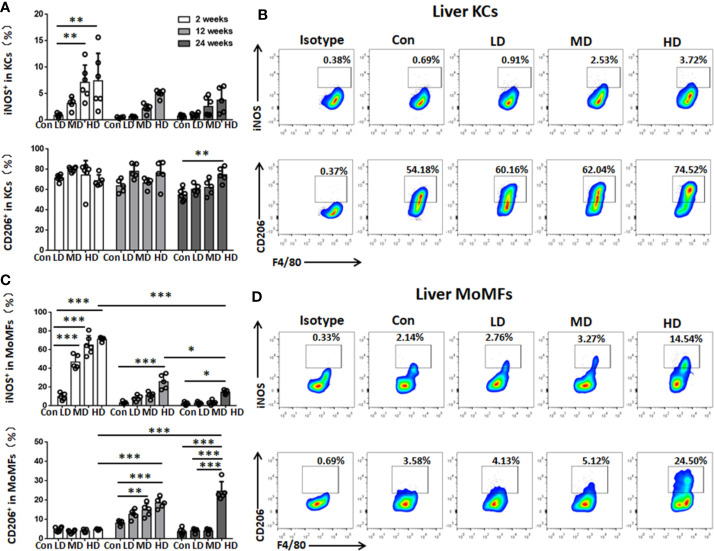
Changes in hepatic macrophage subset polarization in mice infected with different *E multilocularis* PSC inocula during the course of infection. **(A)** The percentage of iNOS^+^ KCs (M1-type) and the percentage of CD206^+^ KCs (M2-type) of the total KCs in the liver. **(B)** Representative flow cytometry plots of intracellular staining of iNOS^+^ and CD206^+^ in KC subsets after 24 weeks of infection. **(C)** The percentage of iNOS^+^ MoMFs (M1-type) and the percentage of CD206^+^ MoMFs (M2-type) of the total MoMFs in the liver. **(D)** Representative flow cytometry plots of intracellular staining of iNOS^+^ and CD206^+^ in MoMF subsets after 24 weeks of infection. Con; LD: 50 PSCs; MD: 500 PSCs; HD: 2000 PSCs. Data are shown as the mean ± standard error of the mean (SEM, five to six mice per group), *p < 0.05, **p < 0.01 and ***p < 0.001.

To characterize the functional phenotype of hepatic macrophages in the livers of mice with different PSC inocula during the time course, we next analyzed the expression of proinflammatory markers (iNOS) and anti-inflammatory markers (CD206) in KCs and MoMFs by flow cytometry analysis. We found that iNOS^+^ KCs (a proinflammatory M1 phenotype) were significantly increased in HD and MD group mice compared to control group mice at 2 weeks, while CD206^+^ KCs (an anti-inflammatory M2 phenotype) were not significantly changed between these groups at 2, 12, and 24 weeks, but the percentage and absolute numbers of CD206^+^ KCs were higher than iNOS^+^ KCs and represented approximately 70% of KCs during the time course (**Figures 4A, B**; **Supplemental Figures S2**, **S3**). The percentage and absolute numbers of iNOS^+^ MoMFs were significantly increased in the HD group compared to the control group mice at 2, 12, and 24 weeks, but gradually decreased from 2 to 24 weeks. In contrast, the percentage of CD206^+^ MoMFs was significantly increased in the HD group compared to the control group mice at 12 and 24 weeks, and the absolute numbers of CD206^+^ MoMFs in the HD group was also significantly increased from 2 to 24 weeks and higher than iNOS^+^ MoMFs at 24 weeks (**Figures 4C, D** **; Supplemental Figures S2**, **S3**).

**Figure 5:**
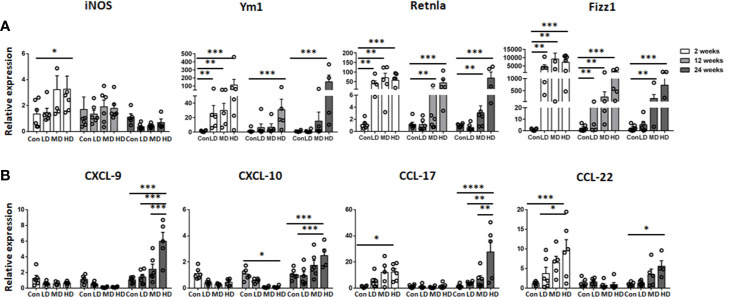
M1 and M2 macrophage-specific marker and chemokine expression in mouse livers infected with different *E multilocularis* PSC inocula during the course of infection. Quantitative RT-PCR analysis for mRNA levels of M1 and M2 macrophage-specific marker and chemokine in whole liver tissues at various time points after infection normalized by comparison to the housekeeping gene GAPDH mRNA. **(A)** Expression of M1 marker (iNOS) and M2 macrophage markers (Ym1, Retnla, Fizz1). **(B)** Expression of M1-related chemokines (CXCL9, CXCL10) and M2-related chemokines (CCL17, CCL22). Con; LD: 50 PSCs; MD: 500 PSCs; HD: 2000 PSCs. Data are shown as the mean ± standard error of the mean (SEM, five to six mice per group), *p < 0.05, **p < 0.01, ***p < 0.001, and ****p < 0.0001.

In addition, we further compared the mRNA expression of M1 (iNOS) and M2 macrophage markers (Ym1, Retnla, and Fizz1) and related chemokines in liver tissues in all the studied groups by qRT-PCR analysis. Correspondingly, our results showed that the expression of iNOS was increased in the HD group compared to the control group at 2 weeks and then declined at 12 and 24 weeks. However, the expression of the anti-inflammatory M2 macrophage markers (Ym1, Retnla and Fizz1) was significantly increased in the HD group compared to the control group and was maintained at a high level during the time course (**Figure 5A**). Moreover, the expression of the M1-related chemokines CXCL9 and CXCL10 was decreased in the LD, MD, and HD groups compared with the control group at 2 and 12 weeks and only increased at 24 weeks. However, the expression of the M2-related chemokines CCL17 and CCL22 was significantly increased in the HD group at 2 and 24 weeks (**Figure 5B**). These results are in line with observations in human AE samples; the hepatic macrophages in liver tissues of murine models showed a predominantly M2 anti-inflammatory phenotype at the chronic stage.

### Depletion of Hepatic Macrophages Impairs Worm Expulsion and Yields High Parasite Burdens

**Figure 6:**
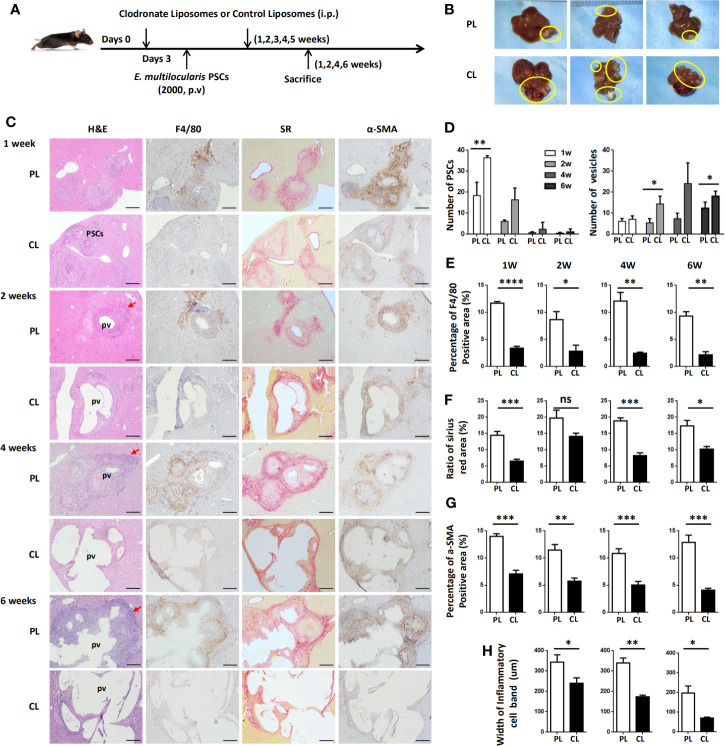
Hepatic macrophage depletion impairs PSC clearance and promotes disease progression in the *E multilocularis*-infected mouse model. **(A)** Protocol for hepatic macrophage depletion. Mice were administered (i.p.) with CL or PL at day 0 before *E multilocularis* infection at day 3 and then administered once a week for 6 weeks. Livers were collected at weeks 1, 2, 4, and 6 after *E multilocularis* infection. **(B)** Representative images of metacestode tissue in the liver from *E multilocularis*–infected mice treated with CLs or PLs (control) after 6 weeks of infection. Metacestode tissues are encircled by the yellow line. **(C)** Representative H&E staining and immunohistochemistry of F4/80, α-SMA, and SR staining of liver sections from *E multilocularis*-infected mice at weeks 1, 2, 4, and 6 after macrophage depletion or control (100× magnification; scale bar, 200 μm). PSCs: protoscoleces. pv: parasitic vesicle. The red arrow indicates the inflammatory cell zone. **(D)** Number of PSCs and parasitic vesicles in the liver from *E multilocularis*-infected mice treated with CL or PL. **(E)** The percentage of positively stained area was calculated to assess the expression of F4/80. **(F)** The ratio of SR staining area was calculated. **(G)** The percentage of positively stained area was calculated to assess the expression of α-SMA. **(H)** Thickness of the inflammatory cell infiltration bands around the metacestode lesions. PSCs: protoscoleces. p.v: portal vein. CL: clodronate-liposomes. PL: phosphate-buffered saline control liposomes. Data are shown as the mean ± standard error of the mean (SEM, four to five mice per group), *p < 0.05, **p < 0.01, ***p < 0.001, ****p < 0.0001. ns, no significance.

To examine whether hepatic macrophages play an important role in the establishment of the *E. multilocularis* metacestode in a mouse model, mice were administered with CL to deplete hepatic macrophages. We first compared the deplete efficiency of hepatic macrophage by i.v. and i.p. injection with CL, finding that no matter which i.v. (about 24 h) or i.p. (about 3 days) with CL, both of the two methods can almost completely deplete KCs, which was consistent with previous report (37), with MoMFs also being largely depleted. After 7 days, new macrophages, especially MoMFs replaced the depleted ones by i.v. By contrast, i.p. with CL can maintain depletion for about 7 days (**Supplemental Figure S4**). Considering our above histopathological and flow cytometric analysis of hepatic macrophage that large number of MoMFs displaying a proinflammatory M1 phenotype were recruited into the liver lesion at early stage (**Figures 2**–**4**), we treated mice i.p. with CL 3 days prior to p.v. injections with PSC and then administered on a weekly basis for 6 weeks to maintain depletion. Control groups were injected with phosphate-buffered saline liposomes (PL) at the same time (**Figure 6A**). After 1, 2, 4, and 6 weeks of PSC inoculation, mice were sacrificed and parasite loads were evaluated.

Through gross observation of the liver surface, we found that hepatic metacestode growth was significantly enhanced in CL-treated mice (**Figure 6B**). We further evaluated the parasite loads by counting the number of infectious foci with PSC or metacestode structure lesions in all liver lobes on H&E-stained sections. We found that mice receiving CL displayed a high number of infectious foci with PSCs compared with mice receiving PL at 1 and 2 weeks, therefore resulting in an increased number of infectious foci with metacestode structure lesions at 4 and 6 weeks (**Figures 6C, D**). IHC analysis confirmed that F4/80^+^ macrophages and CD4^+^ T lymphocytes in the periparasitic infiltration areas were markedly reduced in CL-treated mice compared with PL mice. Additionally, CL treatment markedly reduced liver fibrosis and activated HSC numbers in the liver, as assessed by SR staining and a-SMA immunostaining (**Figures 6C, E–G**; **Supplemental Figure S5**). Moreover, the lesion area in CL-treated mice was significantly larger than that in PL-treated mice. Notably, the metacestode lesions embedded within thin fibrosis tissue and the thickness of the inflammatory cell infiltration bands around the metacestode lesions were thinner in the CL group than in the PL group mice (**Figures 6H**). These data demonstrated that hepatic macrophage depletion led to impaired worm expulsion and yielded a high parasite burden.

## Discussion

Numerous studies on parasite or virus infection and tumor development in the liver have shown that hepatic macrophages are involved in the progression of liver inflammation and fibrosis, and therefore, they play a key role in controlling the pathogenesis of liver disease (38–40). Hepatic macrophages are markedly heterogeneous cell populations, and their phenotypes and functions are likely switching during disease progression (38). Recently, we have shown that the number of macrophages is increased in the liver resections of patients with AE (13), suggesting that hepatic macrophages are involved in the development of AE. However, the characteristics of hepatic macrophage phenotypes and functions during *E. multilocularis* infection, especially at an early stage of infection, have been less studied. In the present study, our data clearly demonstrated that in response to *E. multilocularis* infection, monocyte-derived macrophages were recruited into the liver and skewed from the M1 proinflammatory phenotype at an early stage towards the M2 anti-inflammatory phenotype at a late stage to maintain persistent infection. Furthermore, depletion of liver macrophages by CL before *E. multilocularis* infection reduced liver fibrosis and increased worm burden, suggesting that hepatic macrophages play a dual role during *E. multilocularis* infection, which is critical for *E. multilocularis* clearance in the early stage but also significant for *E. multilocularis* immune escape in the late stage.

Parasite establishment in the host depends on the resolution of inflammation (41). Previous studies reported that microbicidal nitric oxide (NO) produced by M1 proinflammatory macrophages is favorable to kill PSCs of *E. multilocularis in vitro* (42), and *E. multilocularis* laminated-layer components could decrease NO production (43), indicating that *E. multilocularis* larvae have the ability to inhibit M1 proinflammatory macrophages in their struggle for survival. However, it has been reported that M2 anti-inflammatory macrophages contribute to the long-term escape of viruses, parasites, or tumor cells from immune attack, leading to chronic infections and protecting the host from excessive pathology (44, 45). In line with previous studies (12, 13), we showed that increased infiltration of CD68^+^ macrophages was readily identified in the CLT of patients with AE compared to DLT. Moreover, both proinflammatory (S100A9^+^) and anti-inflammatory (CD163^+^) macrophages were detected in larger numbers in CLT than in DLT, and the number of CD163^+^ anti-inflammatory macrophages was higher than that of S100A9^+^ proinflammatory macrophages, indicating that the infiltrated macrophages around the metacestode in the liver predominantly exhibit an M2 anti-inflammatory phenotype that may promote the survival of *E. multilocularis* in the liver organ. Taken together, these findings suggest an important role for infiltrating macrophages in human AE. As patients with AE once diagnosed usually reach the middle or late stage of the disease, it is necessary for us to use animal models to further examine this phenomenon.

The liver location of AE lesions is important for studying the immune response elicited in the liver of *E. multilocularis* infection. Therefore, we used a suitable experimental mouse model by direct injection of precise numbers of *E. multilocularis* PSCs *via* the portal vein, which can mimic the ‘natural’ location (32) and enable us to study the dynamic changes in hepatic macrophage phenotypes and functions during *E. multilocularis* infection. In our study, we not only detected pathological changes and F4/80^+^ macrophage expression in mouse livers from the reported early to middle and later stages of infection (32) but also detected F4/80^+^ macrophage expression from the very early stage of infection (2, 5, 8, and 11 days). Our results showed that at 2 days, many *E. multilocularis* PSCs from the hepatic portal vein inoculum were present and maintained an intact structure in the infected livers, and only a few infiltrating lymphocytes surrounding the PSC inoculum could be observed. From 5 days to 11 days, PSCs could still be observed, and the worm body gradually disintegrated and disappeared. Only a few PSCs were able to develop into *E. multilocularis* metacestodes, which were observed from 2 weeks to 24 weeks after infection (32). Notably, numerous inflammatory cells began to be recruited surrounding the PSC inocula as early as 5 days post infection, and F4/80^+^ macrophages were also markedly increased in the periparasitic inflammatory area at 5 days and peaked at 12 weeks. In addition, accumulative evidence demonstrates that hepatic macrophages consist of liver resident macrophages (KCs) and MoMFs, which are rapidly recruited from the circulation to the injury site through the chemokine receptor CCR2 during acute or chronic injury to the liver (15, 17). In our study, additional immunofluorescence of the infected mouse livers revealed that the accumulated F4/80^+^ macrophages express CCR2, thereby underlining MoMFs recruitment during AE progression. We further used flow cytometry on freshly isolated mouse liver tissues to confirm the hepatic macrophage composition and found that the number of CD11b^hi^ F4/80^int^ MoMFs was significantly increased in *E. multilocularis*-infected mice, especially in HDG, compared to the control group and even exceeded the CD11b^int^F4/80^hi^ KCs at an early stage (2 weeks), indicating that MoMFs are massively attracted to the parasite infection site and represent the dominant hepatic macrophage population during the early infection stage of *E. multilocularis*, which may participate in the early clearance of *E. multilocularis* in mice.

Additionally, we further detected the M1 proinflammatory and M2 anti-inflammatory phenotypes of resident KCs and recruited MoMFs in the liver during different infection times. Our data showed that resident KCs are always dominated by the anti-inflammatory M2 phenotype during the time course. In contrast, recruited MoMFs are predominantly polarized into the M1 proinflammatory phenotype at an early stage and then gradually towards a dominant M2 anti-inflammatory phenotype at a late stage of *E. multilocularis* infection. Correspondingly, qRT-PCR analysis showed that the expression of M2 markers (Ym1, Retnla and Fizz1) was significantly upregulated compared with expression in the control group, exhibiting a similar phenotype in the late stage of hepatic AE. Moreover, we found that the M2 macrophage-related chemokines CCL17 and CCL22, which have been reported to play important roles in attracting T regulatory cells (Tregs) to promote immune tolerance during the tumorigenic process (46), were also significantly upregulated. Our previous studies have shown that Tregs are significantly elevated in livers from infected mice at 24 weeks (32). Therefore, *E. multilocularis* infection-enhanced CCL17 and CCL22 production results in Treg recruitment leading to local immunosuppression, but further research is needed. Taken together, we showed here that *E. multilocularis* infection recruited an increased infiltration of MoMFs to the liver and induced the skewing of their phenotypes from proinflammatory M1 macrophages towards anti-inflammatory M2 macrophages, which favors persistent infection.

Although we have characterized the dynamic changes in hepatic macrophage subsets during *E. multilocularis* infection, the functional role of hepatic macrophages in parasite establishment is still not very clear. Given that clodronate liposomes are now the most effective and widely used method to deplete macrophages (33), we administered CL to the challenged mice every week for 6 weeks. We found that macrophage depletion reduced liver fibrosis and yielded a high parasite burden. These results are consistent with previous reports in the helminth parasites *H. polygyrus* (47) and *N. brasiliensis* (48), showing an impaired ability to expel these two parasites when hosts were depleted of macrophages. Local liver fibrogenesis is actually protective and successfully separates the parasite lesion and limits its continuous growth in the liver. However, liver fibrosis was significantly reduced in the context of macrophage depletion and thereby exacerbated parasite disease. In addition, we found that macrophage depletion also reduced CD4^+^ T lymphocytes infiltration around the lesion. As our recent study have showed that hepatic metacestode growth was significantly enhanced in mice depleted of CD4^+^ T cells (13), indicating that depletion of hepatic macrophages impairs worm expulsion may also be partly due to a decrease in liver infiltration of CD4^+^ T cells, thereby causing the aggressive growth of metacestode. These findings demonstrate that hepatic macrophages play a critical role in host defense against *E. multilocularis* infection in which their early recruitment may favor parasite clearance.

In summary, our study demonstrates that hepatic macrophages, both proinﬂammatory M1 macrophages and anti-inﬂammatory M2 macrophages, participate in the infection progression of *E. multilocularis* in both humans with AE and *E. multilocularis*-infected mice. During the early stage of parasite infection, hepatic macrophages are mainly composed of infiltrated MoMFs and dominated by a proinflammatory M1 phenotype to clear the parasites by producing proinflammatory cytokines. In contrast, chronic *E. multilocularis* infection tends to induce hepatic macrophage polarization towards the anti-inflammatory M2 phenotype by producing anti-inflammatory cytokines to maintain persistent infection. Importantly, depletion of hepatic macrophages impaired worm expulsion and resulted in a high parasite burden. Our results implied that hepatic macrophages may play a dual role in echinococcosis, which promotes early larvae clearance and favors persistent metacestode infection at the late stage. Future studies are needed to explore the exact role of hepatic macrophage subsets and the shift between them, indicating a novel therapeutic approach aimed at targeting hepatic macrophages for patients with AE.

## Data Availability Statement

The raw data supporting the conclusions of this article will be made available by the authors, without undue reservation.

## Ethics Statement

All protocols involving patients with AE were approved by the Ethics Committee of the First Affiliated Hospital of Xinjiang Medical University (Approval No. S20130418-3), and written informed consent was obtained from each subject in accordance with the Declaration of Helsinki (1975) of the World Medical Association. All experimental protocols involving mice were also approved by the Ethics Committee of the First Affiliated Hospital of Xinjiang Medical University (Approval No. 20170809-01).

## Author Contributions

HWa, C-SZ, R-YL, and HWe designed the study. HWa, C-SZ, B-BF, JH, W-DL, X-JB, LL (4th author), and Z-DL performed the experiments and analyses. LL (5th author), AA, and Y-MS contributed to technical or material-related issues. HWa and C-SZ drafted the manuscript. R-YL and HWe supervised the study and critically reviewed the manuscript. All authors contributed to the article and approved the submitted version.

## Funding

This work was supported by the National Natural Science Foundation of China (81860359, 81660342), the Xinjiang Uyghur Autonomous Region Key Laboratory Open Research Program Project (2019D04021), the Postdoctoral Science Foundation of China (2018T111121), and the State Key Laboratory of Pathogenesis, Prevention and Treatment of Central Asia High Incidence Diseases Fund (SKL-HIDCA-2020-5).

## Conflict of Interest

The authors declare that the research was conducted in the absence of any commercial or financial relationships that could be construed as a potential conflict of interest.

## Supplementary Material

Supplementary Figure 1 Gating strategy for the identification of hepatic macrophages. **(A)** Liver lymphocytes were gated**. (B)** Single cells were gated. **(C)** CD45^+^ cells were gated. **(D)** Neutrophils were identified as CD45^+^CD11b^+^ and Ly-6G^+^ cells, and they were excluded from subsequent macrophage gating. **(E)** Cells positive for CD3, CD19, or NK1.1 were excluded from subsequent macrophage gating. **(F)** Resident Kupffer cells (KCs) were selected as CD45^+^Ly-6G^-^CD3^-^CD19^-^NK1.1^-^ and CD11b^int^F4/80^hi^ cells; monocyte-derived macrophages (MoMFs) were selected as CD45^+^Ly-6G^-^CD3^-^CD19^-^NK1.1^-^ and CD11b^hi^F4/80^int^ cells. Representative flow cytometry plots are shown.

Supplementary Figure 2 Absolute numbers analysis of hepatic macrophages from mice infected with *E. multilocularis* during the time course. Con; LD: 50 PSCs; MD: 500 PSCs; HD: 2000 PSCs. Data are shown as the mean ± standard error of the mean (SEM, 5–6 mice per group), *p < 0.05, **p < 0.01, ***p < 0.001, and ****p < 0.0001.

Supplementary Figure 3 Representative flow cytometry plots gated on liver KC and MoMF subsets in mice infected with different *E. multilocularis* PSC inocula during the course of infection. **(A)** Intracellular staining of iNOS^+^ in KCs and MoMFs at 2, 12 and 24 weeks postinfection. **(B)** Intracellular staining of CD206^+^ in KCs and MoMFs at 2, 12 and 24 weeks postinfection. Con; LD: 50 PSCs; MD: 500 PSCs; HD: 2000 PSCs.

Supplementary Figure 4 Flow cytometry analysis of the hepatic macrophage depletion efficiency by intraperitoneal (i.p.) and intravenous (i.v.) administration of clodronate-liposomes.

Supplementary Figure 5 Hepatic macrophage depletion reduces CD4^+^ T lymphocytes accumulation in the inflammatory cell zone around the liver lesions in *E. multilocularis*-infected mice. **(A)** Representative immunohistochemistry of CD4 staining of liver sections from *E. multilocularis*-infected mice at weeks 1, 2, 4, and 6 after macrophage depletion (CL) or control (PL) (scale bar, 200 μm for 100× magnification; 50 μm for 400× magnification). **(B)** The percentage of CD4 positive area was calculated to assess the expression of CD4. Data are shown as the mean ± standard error of the mean (SEM, four to five mice per group), *p < 0.05, **p < 0.01, and ***p < 0.001.
