# Supplementary figures and images for: Dual Role of Hepatic Macrophages in the Establishment of the Echinococcus multilocularis Metacestode in Mice
Source: Front Immunol. 2021 Jan 8;11:600635. doi: 10.3389/fimmu.2020.600635 (PMC7820908; doi:10.3389/fimmu.2020.600635)

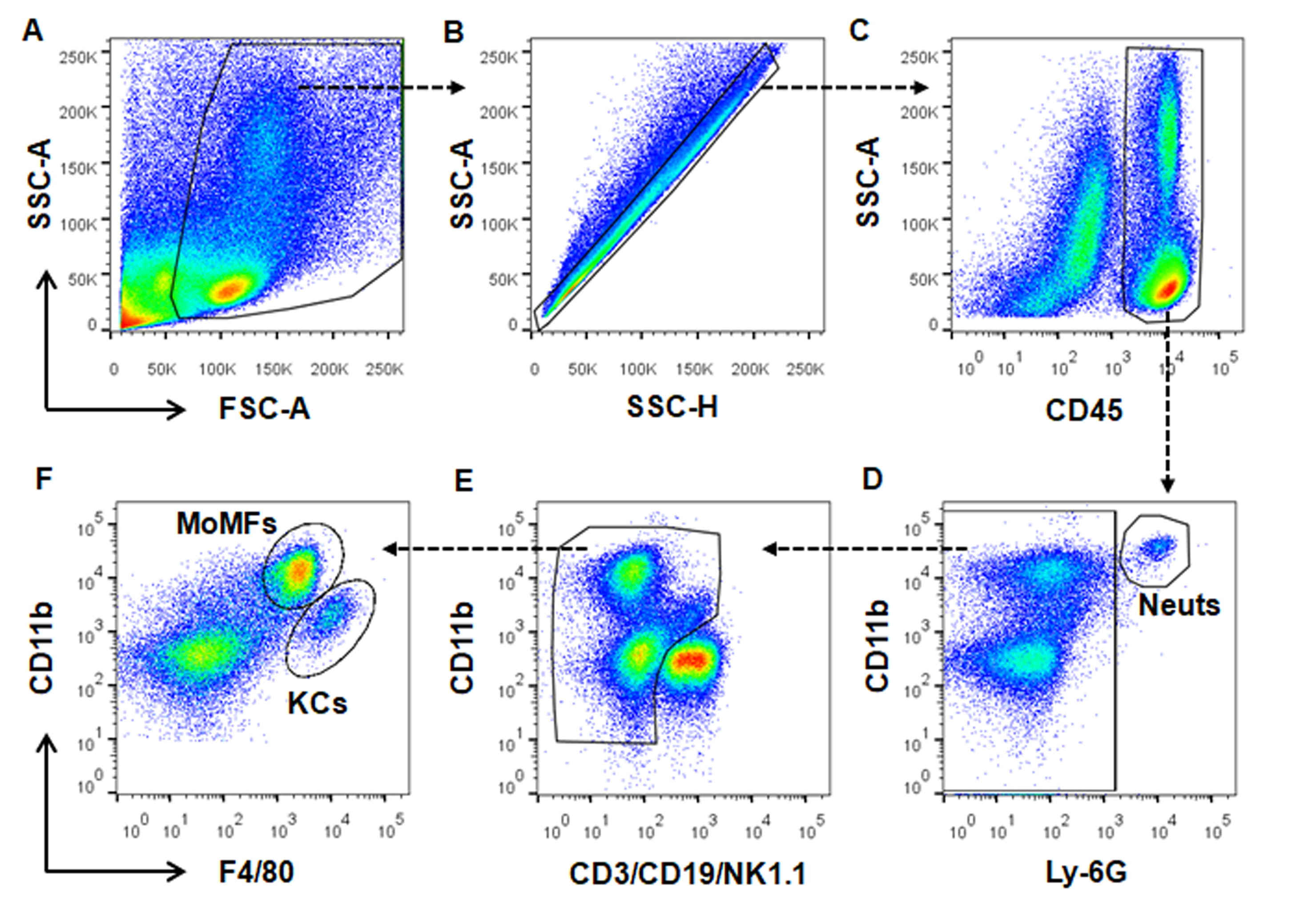

Supplement: Supplementary Figure 1 — Gating strategy for the identification of hepatic macrophages. (A) Liver lymphocytes were gated. (B) Single cells were gated. (C) CD45+ cells were gated. (D) Neutrophils were identified as CD45+CD11b+ and Ly-6G+ cells, and they were excluded from subsequent macrophage gating. (E) Cells positive for CD3, CD19, or NK1.1 were excluded from subsequent macrophage gating. (F) Resident Kupffer cells (KCs) were selected as CD45+Ly-6G-CD3-CD19-NK1.1- and CD11bintF4/80hi cells; monocyte-derived macrophages (MoMFs) were selected as CD45+Ly-6G-CD3-CD19-NK1.1- and CD11bhiF4/80int cells. Representative flow cytometry plots are shown. [file Image_1.tif]

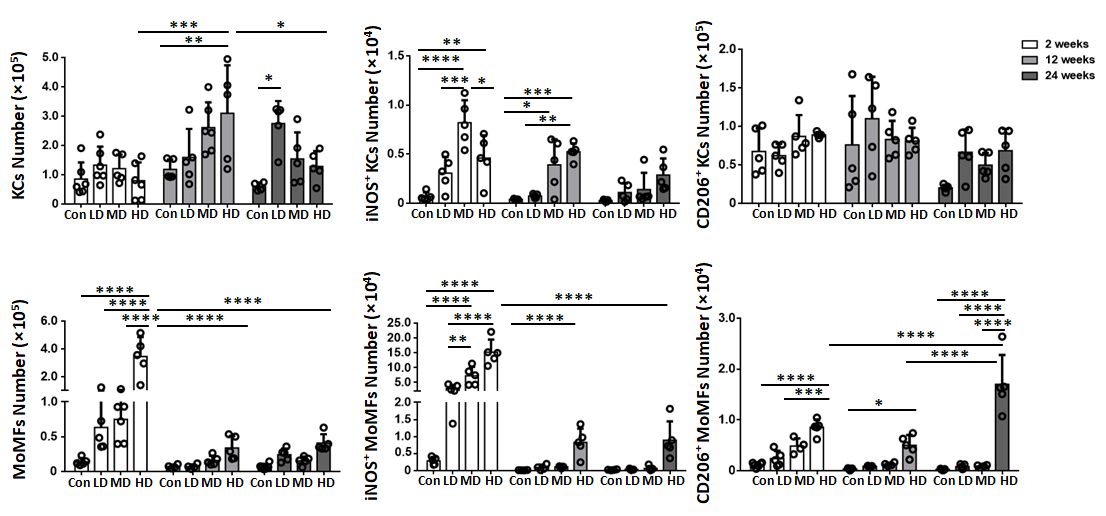

Supplement: Supplementary Figure 2 — Absolute numbers analysis of hepatic macrophages from mice infected with E. multilocularis during the time course. Con; LD: 50 PSCs; MD: 500 PSCs; HD: 2000 PSCs. Data are shown as the mean ± standard error of the mean (SEM, 5–6 mice per group), *p < 0.05, **p < 0.01, ***p < 0.001, and ****p < 0.0001. [file Image_2.tif]

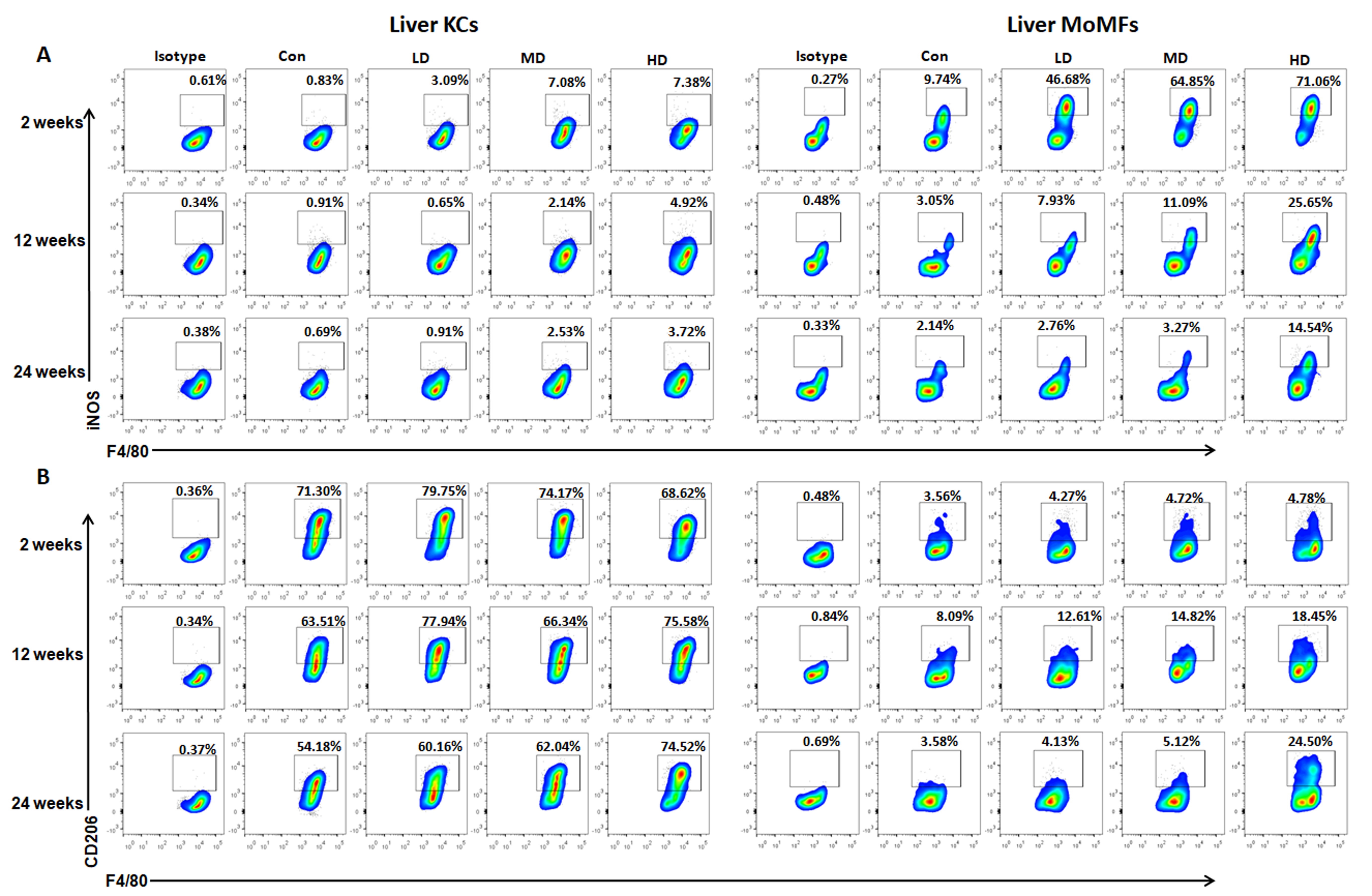

Supplement: Supplementary Figure 3 — Representative flow cytometry plots gated on liver KC and MoMF subsets in mice infected with different E. multilocularis PSC inocula during the course of infection. (A) Intracellular staining of iNOS+ in KCs and MoMFs at 2, 12 and 24 weeks postinfection. (B) Intracellular staining of CD206+ in KCs and MoMFs at 2, 12 and 24 weeks postinfection. Con; LD: 50 PSCs; MD: 500 PSCs; HD: 2000 PSCs. [file Image_3.tif]

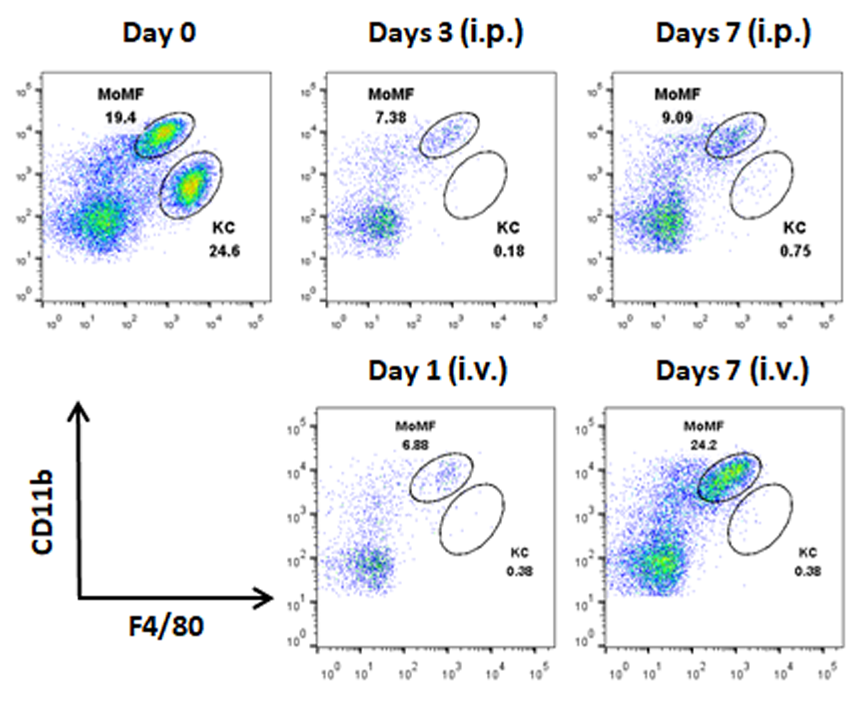

Supplement: Supplementary Figure 4 — Flow cytometry analysis of the hepatic macrophage depletion efficiency by intraperitoneal (i.p.) and intravenous (i.v.) administration of clodronate-liposomes. [file Image_4.tif]

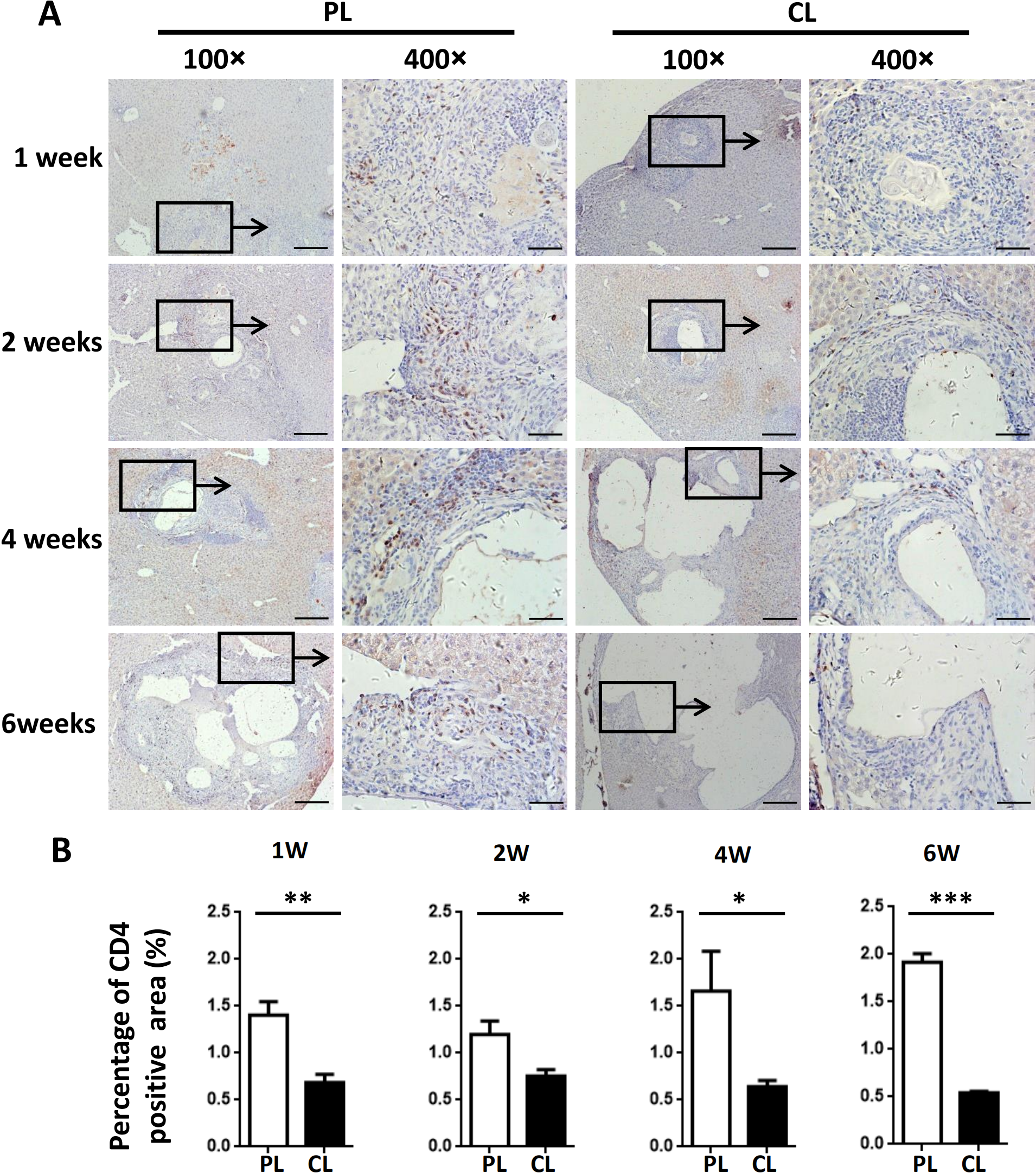

Supplement: Supplementary Figure 5 — Hepatic macrophage depletion reduces CD4+ T lymphocytes accumulation in the inflammatory cell zone around the liver lesions in E. multilocularis-infected mice. (A) Representative immunohistochemistry of CD4 staining of liver sections from E. multilocularis-infected mice at weeks 1, 2, 4, and 6 after macrophage depletion (CL) or control (PL) (scale bar, 200 μm for 100× magnification; 50 μm for 400× magnification). (B) The percentage of CD4 positive area was calculated to assess the expression of CD4. Data are shown as the mean ± standard error of the mean (SEM, four to five mice per group), *p < 0.05, **p < 0.01, and ***p < 0.001. [file Image_5.tif]
