# Supplementary material for: Dual Role of Hepatic Macrophages in the Establishment of the Echinococcus multilocularis Metacestode in Mice
Source: Front Immunol. 2021 Jan 8;11:600635. doi: 10.3389/fimmu.2020.600635 (PMC7820908; doi:10.3389/fimmu.2020.600635)
Supplement: Supplementary file 6 [file Table_1.doc]

**Supporting Table S1. Antibodies for flow cytometry**

| **Reagent or Resource** | **Clone** | **Source** | **Catalog Number** |
| --- | --- | --- | --- |
| Purified anti-mouse CD16/32 |  | BioLegend | Cat# 101302 |
| Anti-mouse CD45- PerCP/Cy5.5 | 30-F11 | BioLegend | Cat# 103132 |
| Anti-mouse CD3-FITC | 17A2 | BioLegend | Cat# 100204 |
| Anti-mouse NK1.1-FITC | PK136 | BioLegend | Cat# 108706 |
| Anti-mouse CD19-FITC | 6D5 | BioLegend | Cat# 115506 |
| Anti-mouse Ly6G-APC-Cy7 | 1A8 | BioLegend | Cat# 127624 |
| Anti-mouse F4/80-PE | BM8 | BioLegend | Cat# 123110 |
| Anti-mouse CD11b Brilliant Violet 650 | M1/70 | BioLegend | Cat# 101239 |
| Anti-mouse CD11b APC | M1/70 | BioLegend | Cat# 101212 |
| Anti-mouse CD206-APC | 15-2 | BioLegend | Cat# 321110 |
| APC Mouse IgG1, κ Isotype Ctrl Antibody |  | BioLegend | Cat# 400119 |
| Anti-mouse iNOS-PE-Cy7 | CXNFT | eBioscience | Cat# 25-5920-82 |
| PE-Cy7 Rat IgG2a kappa Isotype Control |  | eBioscience | Cat# 25-4321-82 |
