# Supplementary material for: Dual Role of Hepatic Macrophages in the Establishment of the Echinococcus multilocularis Metacestode in Mice
Source: Front Immunol. 2021 Jan 8;11:600635. doi: 10.3389/fimmu.2020.600635 (PMC7820908; doi:10.3389/fimmu.2020.600635)
Supplement: Supplementary file 7 [file Table_2.doc]

**Supporting Table S2.** **Primer sequences for qRT-PCR**

| **Gene** | **GenBank Accession** | **Forward primer** | **Reverse primer** |
| --- | --- | --- | --- |
| iNOS | NM_010927.3 | TTCACCCAGTTGTGCATCGACCTA | TCCATGGTCACCTCCAACACAAGA |
| Ym1 | NM_009892.3 | TCTCTACTCCTCAGAACCGTCAGA | GATGTTTGTCCTTAGGAGGGCTTC |
| Fizz 1 | NM_181596.4 | TACTTGCAACTGCCTGTGCTTACT | TATCAAAGCTGGGTTCTCCACCTC |
| Retnla | NM_020509.4 | CTGGATTGGCAAGAAGTTCC | CCCTTCTCATCTGCATCTCC |
| CXCL9 | NM_008599.4 | TCTCGGACTTCACTCCAACACA | ACTCCACACTGCTGGAGGAAGA |
| CXCX10 | NM_021274.2 | CCGTCATTTTCTGCCTCATCC | CCCTATGGCCCTCATTCTCA |
| CCL17 | NM_011332.3 | AGTGCTGCCTGGATTACTTCAAAG | CTGGACAGTCAGAAACACGATGG |
| CCL22 | NM_009137.2 | TAACATCATGGCTACCCTGCG | TGTCTTCCACATTGGCACCA |
| GAPDH | NM_001289726.1 | CATGGCCTTCCGTGTTCCTA | CCTGCTTCACCACCTTCTTGAT |
